# Insights Into Late‐Onset Rheumatoid Arthritis (LORA): Characteristics (Clinical and Imaging), Comorbidities, and Therapeutic Targets

**DOI:** 10.1111/ggi.70399

**Published:** 2026-02-20

**Authors:** Fausto Salaffi, Marina Carotti, Sonia Farah, Francesca Bandinelli, Luca Ceccarelli, Andrea Di Matteo, Marco Di Carlo

**Affiliations:** ^1^ Rheumatology Unit, “Carlo Urbani” Hospital, Dipartimento di Scienze Cliniche e Molecolari, Università Politecnica Delle Marche Jesi, Ancona Italy; ^2^ Clinica di Radiologia, Ospedali Riuniti di Ancona, Università Politecnica Delle Marche Ancona Italy; ^3^ Rheumatology Department San Giovanni di Dio Hospital, USL Tuscany Center Florence Italy; ^4^ Pediatric and Adult Cardiothoracic and Vascular, Oncohematologic and Emergency Radiology Unit, IRCCS Azienda Ospedaliero‐Universitaria di Bologna Bologna Italy; ^5^ Leeds Institute of Rheumatic and Musculoskeletal Medicine, University of Leeds Leeds UK

**Keywords:** comorbidities, frailty, late‐onset rheumatoid arthritis, sarcopenia, treatment

## Abstract

Late‐onset rheumatoid arthritis (LORA) is defined as rheumatoid arthritis (RA) manifesting after the age of 65 years, although the terminology remains somewhat ambiguous. With the advent of a super‐aging society and extended life expectancies, a significant increase in the incidence of LORA is anticipated. In comparison to young‐onset RA (YORA), LORA is predominantly characterized by a higher incidence of acute onset, augmented disease activity and constitutional symptoms, a propensity for systemic manifestations, increased erythrocyte sedimentation rate at disease onset, reduced seropositivity, a predilection for involvement of large and proximal joints with symptoms resembling polymyalgia rheumatica, a higher frequency of erosive disease, and a more evenly distributed gender ratio. Elderly individuals, particularly those with multimorbidity and on multiple medications (polypharmacy), are at an elevated risk of developing geriatric syndromes, including sarcopenia and frailty. The response to TNF inhibitors in elderly individuals with RA is generally comparable to that in younger patients, though it may be slightly diminished. The duration of the disease appears to have a more pronounced impact on outcomes than the patient's age. For the management of LORA, it is critical to adopt a patient‐specific approach. Non‐frail LORA patients who are otherwise aging healthily should receive aggressive treat‐to‐target management. Conversely, in pre‐frail and frail patients, the therapeutic focus should be on averting the progression of irreversible geriatric conditions. The confluence of multimorbidity, polypharmacy, and geriatric syndromes in this patient population necessitates a tailored therapeutic approach to maintain patient autonomy and functional status.

## Graphical abstract

The main features of late‐onset rheumatoid arthritis (LORA), manifesting after age 65, are acute onset, higher disease activity, systemic involvement, and less presence of autoantibodies. Management requires individualized strategies, balancing aggressive treat‐to‐target therapy in robust patients with frailty‐focused approaches to prevent geriatric syndromes, considering comorbidities and polypharmacy.

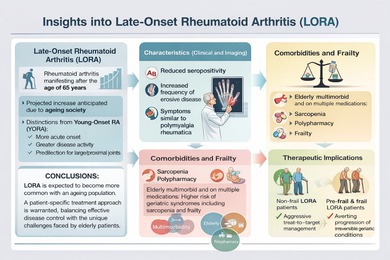

## 1 Introduction

Italy has the second‐highest proportion of elderly individuals in Europe, following Germany. In 2013, over 12 million Italians (about 21% of the population) were aged 65 or older. By early 2018, the average age in Italy surpassed 45 years. This aging trend, seen across Europe, correlates with a rise in chronic age‐related conditions, such as musculoskeletal disorders (MSDs) [1, 2]. After adjusting for gender, approximately 13 million Italians—25% of adults—have experienced or are living with MSDs [2].

**FIGURE 1:**
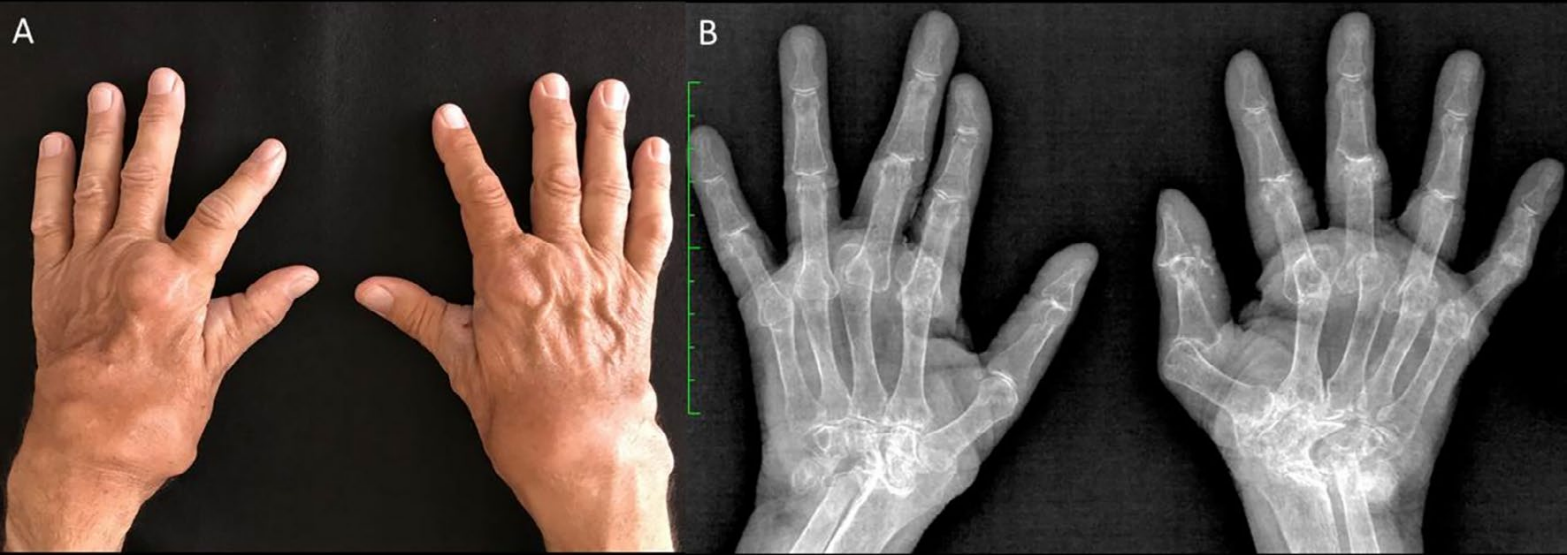
Rheumatoid arthritis. Illustrative case highlighting the burden and disability that rheumatoid arthritis, although of young‐onset, can cause in older adults. This case involves a 70‐year‐old man with a disease duration of over 30 years. The patient exhibits high titers of rheumatoid factor and anti‐cyclic citrullinated peptide antibodies, and the disease has been persistently perceived as difficult‐to‐treat and multi‐refractory. (A) Clinical image of the hands and wrists shows marked swelling of the wrists and metacarpophalangeal joints. (B) Radiograph of the hands and wrists reveals advanced and destructive joint damage, including wrist arthritis (carpitis), erosions, and subluxations.

Rheumatoid arthritis (RA), a chronic autoimmune disease, involves persistent joint inflammation, bone erosion, and impaired joint function, severely affecting physical abilities and health‐related quality of life (HRQoL) [3, 4, 5, 6]. Among inflammatory rheumatic diseases, RA is linked to the highest disability burden [7, 8] (Figure 1).

Although RA can develop at any age, its incidence rises significantly after 60 and plateaus after 80 [9, 10]. RA onset after age 65 is termed late‐onset RA (LORA), while onset at younger ages is referred to as young‐onset RA (YORA). Although other definitions may also be used, among which the most common is elderly onset, LORA represents the terminology most widely accepted by scientific societies [11]. The application of a 65‐year cut‐off as the age of onset distinguishing LORA is itself arbitrary. In this regard, there is ongoing debate, with proposals advocating for a younger age threshold (e.g., from 60 years onward) or for definitions based on specific clinical characteristics [11, 12, 13]. However, about 30% of RA cases occur in people over 60, with increasing prevalence into the eighth decade [12]. This is due to both longer life expectancy and rising RA incidence in older adults. A large Japanese study found the peak age of RA onset shifted from 50–59 to 60–69 over the past decade [14].

RA patients are seven times more likely to experience disability than age‐ and gender‐matched peers [15]. Treating RA in the elderly is challenging due to added risks from age‐related conditions like depression, frailty, cognitive decline, falls, malnutrition, and overall weakness [16, 17].

This narrative review outlines the main pathogenetic, clinical, and therapeutic aspects of LORA, along with its common comorbidities.

## 2 Inflammaging and Rheumatoid Arthritis

In 2000, Franceschi et al. introduced the term inflammaging—a chronic, sterile, low‐grade inflammatory state linked to aging and increased risk of age‐related diseases [18]. It results from impaired clearance of cellular debris and self‐molecules, triggering autoimmune responses. This highlights inflammation as a key factor in aging and its related conditions [19, 20].

Aging alters both innate and adaptive immunity. The innate system becomes overactive, fueling chronic inflammation and comorbidities [21, 22], while the adaptive system deteriorates, reducing immune tolerance and increasing autoimmune disease risk [23, 24, 25]. Age‐related immune changes include T‐cell alterations, weakened responses, apoptosis defects, cytokine imbalance, and diminished antigen presentation—leading to lower protective responses and increased autoimmunity [26].

Self‐tolerance also declines with age. Thymic involution reduces T‐cell proliferation, cytokine production, and post‐vaccination antibody synthesis. In LORA, elevated interleukin‐6 (IL‐6) levels correlate with dehydroepiandrosterone and androstenedione production, contributing to its acute onset [27]. Punzi et al. found higher IL‐6 in LORA synovial fluid than in YORA, though IL‐1 and IL‐8 levels were similar [28].

Immunopathological differences exist between YORA and LORA. Gamerith et al. reported a higher prevalence of rheumatoid factor (RF) in YORA, due to a greater ratio of antigen‐bound to free anti‐IgG‐Fab antibodies (aFab), suggesting age‐specific immune mechanisms in RA development [29].

## 3 Clinical Features of LORA

Recent studies report greater disease activity and severity in LORA compared to YORA, with worse clinical, functional, and radiographic outcomes [30]. LORA more often involves both large and small joints at onset, with higher disease activity scores, elevated erythrocyte sedimentation rate (ESR) and C‐reactive protein (CRP), and more frequent bone erosions in early stages [31] (Table 1).

**TABLE 1:** Comparison of main characteristics of late‐onset rheumatoid arthritis (LORA) and young‐onset rheumatoid arthritis (YORA).

Characteristics	LORA	YORA
Onset age	After 65 years	30–50
Prevalence	2%	0.5%–1%
Female/male ratio (gender ratio)	2/1	3/1
Disease onset	Acute and infectious‐like	Gradual
Number of joints involved	Polyarticular (may be oligoarticular)	Polyarticular
Characteristics of joint involvement	Large/proximal joint	Small joints of the hands and feet
PMR symptoms	More frequent	Less frequent
Systemic manifestations (fatigue, weight loss)	More prominent	Less prominent
Rheumatoid factor positivity	Lower incidence	Higher incidence
ACPA positivity	Lower incidence	Higher incidence
Bone erosions	More frequent	Frequent
Elevated ESR/CRP	More frequent	Frequent
Higher DAS28, CDAI, SDAI	More frequent	Frequent
Clinical patterns	Classical RA, PMR like, RS3PE like	Classical RA
Comorbidity/polyfarmacy/frailty	More frequent	Less frequent
Prognosis and outcome	Worse	Good or worse

Abbreviations: ACPA, anti‐citrullinated protein antibodies; CDAI, clinical disease activity index; CRP, C‐reactive protein; DAS28, 28‐joint disease activity score; ESR, erythrocyte sedimentation rate; LORA, late‐onset rheumatoid arthritis; PMR, polymyalgia rheumatica; RS3PE, remitting seronegative synovitis with pitting edema; SDAI, simplified disease activity index; YORA, young‐onset rheumatoid arthritis.

LORA is generally associated with lower serum levels of anti‐citrullinated protein antibodies (ACPA). A study that stratified patients with RA by age groups demonstrated a significant reduction in both RF and ACPA titers with increasing age at diagnosis, concluding that age itself is a predictor of seronegativity in female, normal‐weight, and non‐smoking individuals [32].

Despite the lower prevalence of ACPA, some studies identify a higher degree of erosions in patients with LORA. Sugihara et al. identified predictors of radiographic progression at 1 year in LORA, including ACPA positivity, baseline DAS28‐ESR, baseline erosion score, lack of EULAR response at 12 weeks, and failure to reach low disease activity (LDA) at 24 weeks [33].

Over 25% of ACPA‐positive LORA patients developed bone erosions despite achieving remission by clinical disease activity index (CDAI) or simplified disease activity index (SDAI) at 1 or 2 years—compared to under 10% in YORA—suggesting these remission criteria may not fully prevent erosions in ACPA‐positive LORA [32].

Greater joint damage and functional decline in LORA may partly stem from age‐related comorbidities, which are more common in LORA than in YORA. These include lung disease, osteoporosis, cardiovascular disease, cancer, body composition changes, and neuropsychiatric disorders [34, 35, 36]. The interplay between RA and immune senescence likely exacerbates these outcomes.

### 3.1 Differential Diagnosis for LORA

Given the prevalence of both inflammatory and noninflammatory rheumatic diseases in older adults, a thorough differential diagnosis is essential for LORA [37] (Table 2). Inflammatory arthritis may signal other systemic conditions. Symptoms like sicca syndrome, skin changes, sclerodactyly, parotid swelling, Raynaud's phenomenon, or myopathy should prompt evaluation for diseases such as systemic sclerosis, dermatomyositis, or Sjögren's syndrome. Assessment should include a full physical exam and relevant lab tests.

**TABLE 2:** Conditions in differential diagnosis with late‐onset rheumatoid arthritis (LORA).

Hand osteoarthritis
Polymyalgia rheumatica
RS3PE syndrome
Crystal arthritides (gout, CPPD)
Spondyloarthropaties
Connective tissue diseases
Systemic vasculitides
Hypertrophic osteoarthropathy
Sarcoidosis
Infectious arthritis (viral and bacterial infections)
Paraneoplastic syndromes
Multiple myeloma

Abbreviations: CPPD, calcium pyrophosphate dehydrate crystal deposition disease; RS3PE, remitting seronegative synovitis with pitting edema.

**FIGURE 2:**
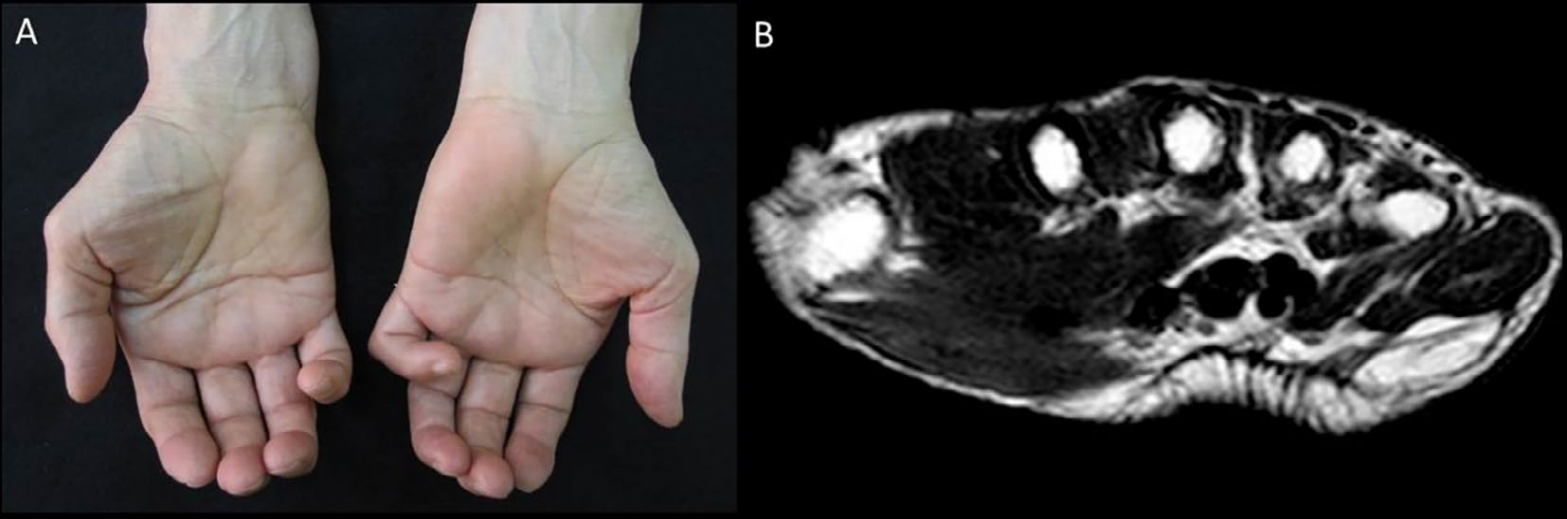
Paraneoplastic syndrome. Palmar fasciitis with polyarthritis (PFPA) associated with ovarian cancer. A 68‐year‐old woman with a 4‐month history of spontaneous left‐hand digital contractures. (A) Clinical image showing the palmar surfaces was hard and wooden feeling with deepening of the palmar crease causing a “groove sign.” (B) Axial T1‐weighted images of the hand of the patient showed an ill‐defined area of low signal intensity within the subcutaneous tissues of the palmar aspect of the hand consistent with diffuse fibromatosis.

In older patients, RA symptoms may indicate a paraneoplastic syndrome [38]. Cancer‐related arthritis often mimics RA but can resolve after tumor treatment. Multicentric reticulohistiocytosis resembles RA with severe arthritis and distinctive facial and periungual papules; it has strong ties to malignancy. Palmar fasciitis, linked to ovarian and breast cancers, causes rapid flexion contractures and painful hand swelling. MRI shows fascial and tendon thickening, especially in muscles like the flexor carpi ulnaris (Figure 2). Amyloid arthropathy, a possible sign of multiple myeloma, may present as RA‐like arthritis, carpal tunnel syndrome, or shoulder enlargement.

**FIGURE 3:**
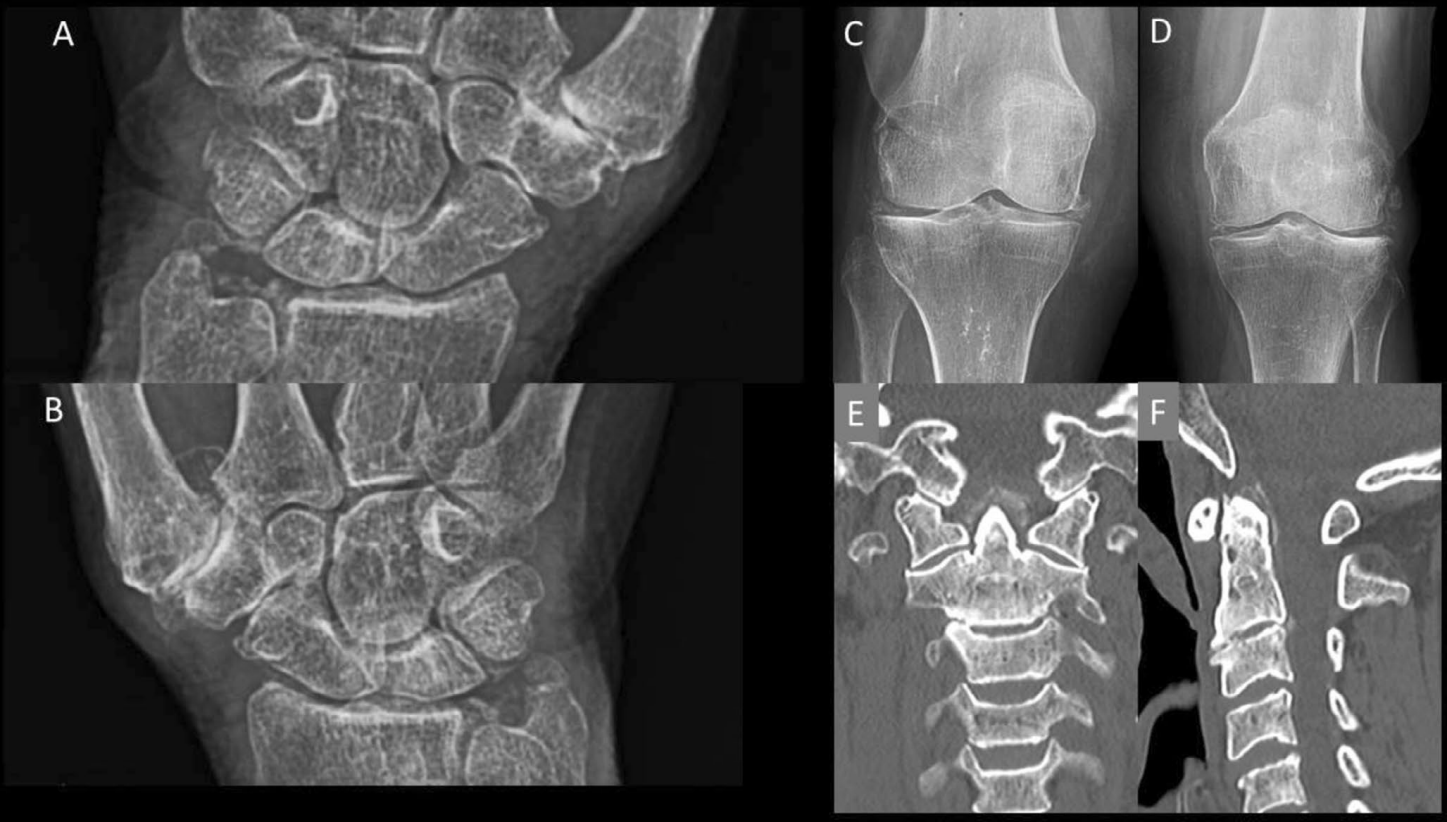
Calcium pyrophosphate dihydrate (CPPD) crystal deposition disease. An 85‐year‐old woman with recent onset of polyarthritis and episodes of inflammatory cervical pain. (A, B) Radiographic images of the wrists reveal the presence of calcification of the triangular fibrocartilage complex. (C, D) Radiographic images of the knees show meniscal chondrocalcinosis. (E, F) Computed tomography multiplanar reconstructions images obtained in the coronal (E) and sagittal (F) planes demonstrate calcific deposits in the ligaments surrounding the odontoid process of the axis, consistent with crowned dens syndrome.

Gout, common in the elderly, can mimic RA when chronic, with erosive deformities and persistent synovitis. Unlike RA's marginal erosions and osteopenia, gout shows distant, sclerotic erosions [39, 40]. Calcium pyrophosphate dihydrate (CPPD) crystal deposition disease, increasingly common with age, causes inflammatory arthritis, especially in knees, shoulders, and wrists [41, 42] (Figure 3). Diagnosis relies on crystal identification via tissue, imaging, or synovial fluid analysis.

**FIGURE 4:**
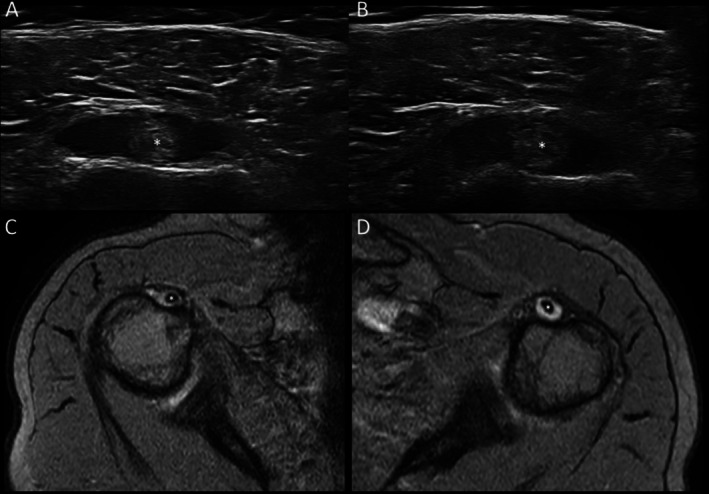
Polymyalgia rheumatica. An 80‐year‐old man with recent onset of inflammatory pain involving the shoulder and pelvic girdles, accompanied by a biological inflammatory syndrome. Transverse ultrasound (US) (A, right and B, left) and axial T2‐weighted gradient echo magnetic resonance images (MRI) (C, right and D, left) sequences of shoulders showing a representative example of bilateral long head of the biceps tenosynovitis. Notably, the widening of both tendon sheaths is due to anechoic (US) or hyperintense (MRI) signals, indicating an abnormal accumulation of synovial fluid. Asterisk indicates tendon of long head of the biceps.

**FIGURE 5:**
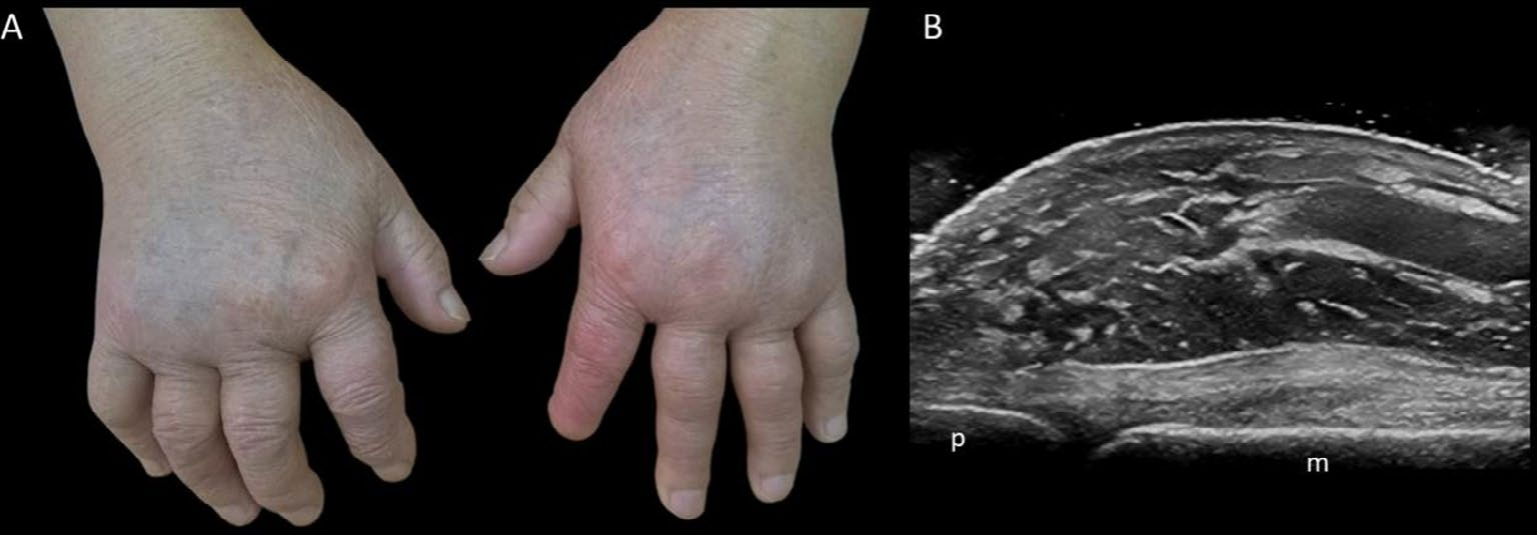
Remitting seronegative symmetrical synovitis with pitting edema (RS3PE) syndrome. An 85‐year‐old woman with recent onset of inflammatory pain associated with swelling of the hands and feet. (A) Clinical image showing the presence of bilateral pitting edema on the dorsum of the hands. (B) Ultrasound image (linear probe, 6–18 MHz) at the level of the third metacarpophalangeal joint of the right hand, demonstrating subcutaneous soft tissue swelling in the absence of clear signs of joint inflammation. m, metacarpal bone; p, base of the proximal phalanx.

LORA may have distinct pathophysiology from YORA, showing greater overlap with polymyalgia rheumatica (PMR) and remitting seronegative symmetrical synovitis with pitting edema (RS3PE) syndrome (Table 3). PMR and LORA can both involve shoulder inflammation. PMR presents with shoulder/pelvic girdle pain and stiffness, lacks ACPA, rheumatoid nodules, and small joint involvement [43, 44], and affects extracapsular structures such as bursae and tendons [45, 46] (Figure 4). LORA typically involves large joints and is ACPA‐positive. Imaging evidence of synovitis supports LORA; bursitis or peritendinitis favors PMR. RS3PE, affecting those over 60, features acute symmetrical synovitis, hand/ft edema, negative RF and ACPA, no erosions, and strong steroid response with potential spontaneous remission [47, 48, 49] (Figure 5).

**TABLE 3:** Different clinical and laboratory features of late‐onset rheumatoid arthritis (LORA), polymyalgia rheumatica (PMR), and remitting seronegative synovitis with pitting edema (RS3PE).

Characteristics	LORA	PMR	RS3PE
Involvement of the shoulder and pelvic girdles	+	+++	−
Peripheral joint symptoms	+++	+	+++
Tenosynovitis/peritendinitis	++	+/−	+++
Pitting edema on the back of the hands	+	+	+++
Rheumatoid factor presence	+/−	−	−
Anti‐citrullinated protein antibodies presence	+/−	−	−
High erythrocyte sedimentation rate/C‐reactive protein	+	+++	+

*Note:* −, absent; +, occasional; ++, frequent; +++, very frequent.

Abbreviations: LORA, late‐onset rheumatoid arthritis; PMR, polymyalgia rheumatica; RS3PE, remitting seronegative synovitis with pitting edema.

**FIGURE 6:**
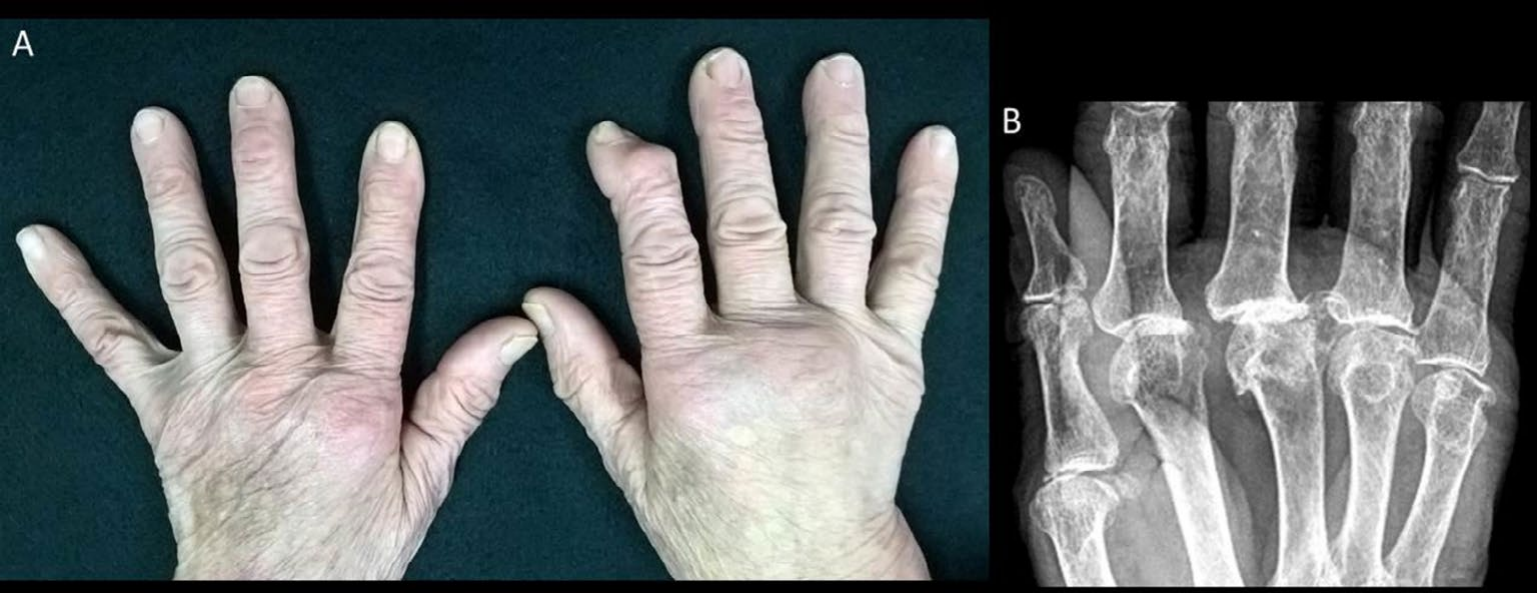
Hand osteoarthritis. A 79‐year‐old man presenting with predominantly mechanical pain and ‘hard’ swelling of the second and third metacarpophalangeal (MCP) joints of the right hand. The clinical picture, initially interpreted as seronegative rheumatoid arthritis, was refractory to multiple lines of treatment with both conventional synthetic and biologic disease‐modifying antirheumatic drugs. Subsequent re‐evaluation—including radiographic, clinical (noting the patient's occupational history as a plumber), and laboratory assessments—allowed for the exclusion of rheumatoid arthritis, supporting a diagnosis of hand osteoarthritis. (A) Clinical image showing swelling of the second and third right MCP joints. (B) Radiographic detail of the right metacarpal region demonstrating osteoarthritic changes (marked joint space narrowing, particularly at the third MCP joint, osteophyte formation, and subchondral sclerosis).

Hand osteoarthritis (HOA) usually causes mild symptoms (Figure 6), but its erosive form—characterized by central erosions and inflammation—can mimic RA [50, 51, 52, 53]. Erosive HOA affects 2.8% of those over 55 and 10.2% of patients with symptomatic HOA, surpassing RA prevalence in this group [54, 55].

## 4 Multimorbidity, Polypharmacy and Frailty

LORA is frequently accompanied by multimorbidity, polypharmacy, and frailty. RA in older adults accelerates immunosenescence, increasing comorbidities such as cardiovascular disease, interstitial lung disease (ILD), infections, cancer, depression, and cognitive decline [56]. The term multimorbidity is preferred over comorbidity to reflect complex interactions among conditions [57]. Distinguishing age‐ versus RA‐related diseases is difficult, but both limit treatment options due to contraindications [58]. In the Netherlands, 60% of people over 60 have ≥ 2 conditions, and polypharmacy risks grow with age and multimorbidity.

LORA patients often experience overlapping rheumatic and non‐rheumatic diseases, complicating care [12, 59]. Common coexisting conditions include diabetes, thyroid disease, metabolic syndrome, osteoarthritis, osteoporosis, sarcopenia, depression, and hypertension. This complexity calls for a multidisciplinary approach.

Polypharmacy, defined as ≥ 5 medications, is common in LORA due to multimorbidity and treatment needs. It is linked to falls, disability, hospitalization, and mortality [60, 61].

A particular mention should be made of treatment with glucocorticoids, which are frequently prescribed within polypharmacological treatment regimens, co‐responsible for significant comorbidities such as cardiovascular disease, Type II diabetes mellitus, infections, and osteoporosis, to name only the main ones. It has been shown that in steroid‐naïve patients, prednisone doses greater than 5 mg/day, higher cumulative doses, and longer treatment duration are associated with an increased risk of cardiovascular events [62]. Low‐dose treatment (< 10 mg/day of prednisone) results in an increased risk of serious infectious events in one out of 83 patients during the first 6 months of therapy [63].

Over half of those aged 65+ are prescribed > 6 medications; 20% receive inappropriate prescriptions [64, 65]. Polypharmacy has been reported in ~34% of RA patients and ~50% at treatment onset in RA and PsA cohorts [60, 66, 67].

Frailty, a syndrome of decreased physiological reserves and increased vulnerability, is gaining attention in rheumatology [68, 69, 70]. It increases risks of falls, disability, post‐surgical mortality, and graft rejection [71, 72, 73, 74]. In RA, frailty prevalence is 27.6%, with pre‐frailty at 32.4%, similar to older populations [75, 76, 77]. It correlates with comorbidities, age, and disease activity [69]. Contributing factors include inflammation, hormonal decline, fatigue, pain, depression, cognitive impairment, and sarcopenia [78, 79, 80, 81, 82]. Cytokines may directly impact the CNS, worsening fatigue [83].

**FIGURE 7:**
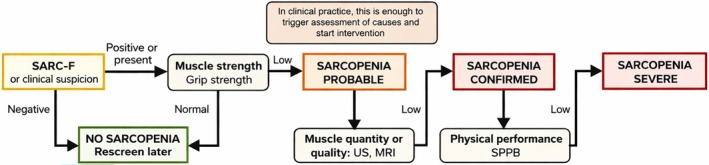
Sarcopenia assessment. Flowchart for case‐finding, making a diagnosis and quantifying severity in rheumatological practice (adapted from [89]).MRI, magnetic resonance imaging; SARC‐F, Strength, Assistance with walking, Rise from a chair, Climb stairs, Fall and calf circumference; SPPB, Short Physical Performance Battery; US, ultrasound.

**FIGURE 8:**
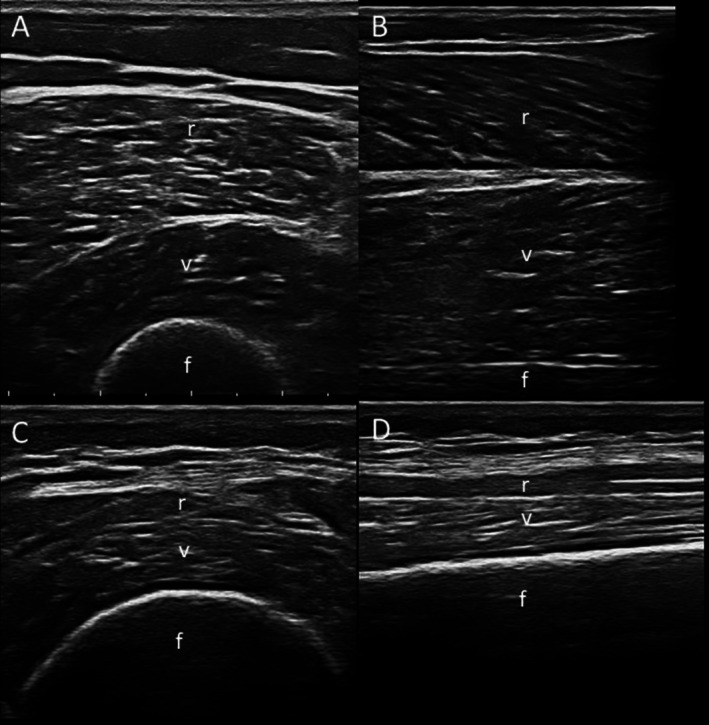
Sarcopenia assessment using ultrasound (US). Images acquired with a linear probe (3–13 MHz). Comparison between a healthy subject (40‐year‐old woman who regularly engages in sports activities) and a sarcopenic subject (88‐year‐old woman with rheumatoid arthritis). Transverse (A, C) and longitudinal (B, D) US images of the anterior thigh compartment muscles (rectus femoris, vastus intermedius). Images (A) and (B) correspond to the healthy subject; images C and D correspond to the sarcopenic subject. (C) and (D) images show a reduced thickness of muscles bellies, and an increased muscles echogenicity compared to (A) and (B) (i.e., Grade III of the Heckmatt scale [i.e., marked increased muscle echo with reduced bone echo] vs. Grade I of the Heckmatt scale [i.e., normal hypoechoic muscle], respectively).f, femur; r, rectus femoris; v, vastus intermedius.

**FIGURE 9:**
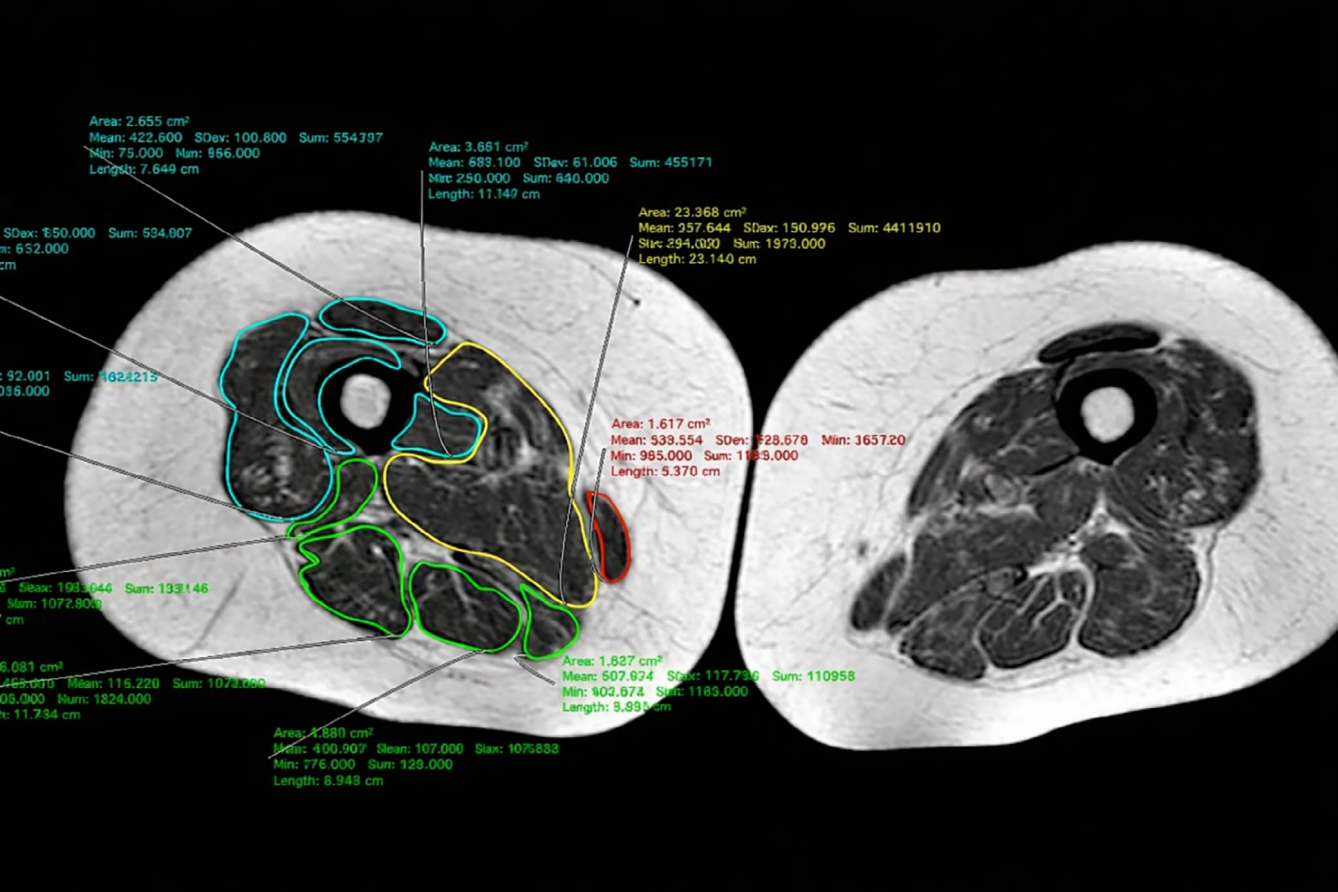
Sarcopenia assessment using magnetic resonance imaging (MRI). Example of segmentation of the mid‐thigh muscles on a single MRI image in a sarcopenic patient using the software Horos.

Sarcopenia, defined in 2018 by European Working Group on Sarcopenia in Older People 2 (EWGSOP2) as loss of muscle mass and strength, increases risks of falls and frailty [84]. Its prevalence in RA is ~31% [85], and it is strongly linked to aging and inflammation [84, 86, 87, 88]. Diagnostic tools include dual energy x‐ray absorptiometry (DXA), bioelectrical impedance analysis (BIA), ultrasound (US), and magnetic resonance imaging (MRI) (Figure 7) [89, 90, 91, 92, 93]. US can assess muscle echogenicity via grayscale pixel analysis [92, 93] (Figure 8), while MRI provides detailed images of muscle mass, quality, and composition (e.g., myosteatosis, myofibrosis) [94, 95]. Automated segmentation of muscles on T1‐weighted MRI can aid accurate sarcopenia detection (Figure 9) [93].

To assess frailty in RA, we developed the Comprehensive Rheumatologic Assessment of Frailty (CRAF), based on the Frailty Index (FI) and cumulative deficit models [96, 97, 98]. Variables for CRAF were selected using the Delphi method [99]. Key domains include nutrition, muscle strength, fall history, comorbidities, polypharmacy, social activity, pain, exhaustion, physical function, and depression.

### 4.1 Cardiovascular Risk

Increased cardiovascular risk in LORA is attributed to a combination of traditional cardiovascular risk factors, the inflammatory nature of arthritis and atherosclerosis, and the use of non‐steroidal anti‐inflammatory drugs (NSAIDs) and steroids [58]. Additionally, patients with LORA display significantly higher body mass index (BMI), blood creatinine and uric acid levels, and worse metabolic indices compared to those with YORA [59]. The prevalence of ischemic heart disease and heart failure is notably higher in the RA population than in the general population, resulting in increased mortality rates [100, 101].

A population‐based study comparing RA patients with non‐RA individuals of similar demographics revealed that the RA group had a 3.17‐fold increased risk of myocardial infarction leading to hospitalization [102]. Moreover, the incidence of heart failure in RA patients is approximately double that of the non‐RA population [101]. The heightened risk of cardiovascular disease associated with RA seems to be primarily concentrated among individuals who are positive for RF. Aging is identified as a risk factor for ischemic heart disease, with age‐related changes in the innate immune system, characterized by elevated levels of proinflammatory cytokines and CRP, potentially explaining this association [103]. Active RA contributes to this inflammatory state. Chronic inflammation is recognized as a partial mediator of the increased cardiovascular risk in RA, yet conventional cardiovascular risk factors also play significant roles [104].

### 4.2 Risk of Infections

Patients with LORA often experience low mobility, which is a significant risk factor for infections, particularly urogenital and respiratory infections. Additionally, the use of immunomodulatory therapy further escalates the risk of infections in RA patients [58]. Due to the immunomodulatory effects of RA itself or its associated comorbidities, patients are at a higher risk of infections compared to other members of the same community [105]. Moreover, RA treatments can suppress the immune system, augmenting the risk of infection in these patients [106]. Notably, age has been incorporated as an independent risk factor for serious infection in RA patients [107].

### 4.3 Lung Disease

Pulmonary complications associated with RA represent one of the most prevalent extra‐articular manifestations of the disease, encompassing conditions such as pulmonary nodules, pleural effusion, bronchiectasis, and particularly ILD [108, 109]. Additionally, RA patients are susceptible to secondary lung complications arising from immunosuppressive therapy, which can include drug toxicity or opportunistic infections [110]. In our studied cohort of RA patients, we observed an ILD prevalence of 19.2% [111], with various studies reporting a prevalence range from 4% to 68%. Factors such as older age, older age at RA onset, high ACPA titer, and a history of smoking have been independently associated with a diagnosis of RA‐ILD [112, 113, 114]. Our study corroborated these findings, underscoring the link between older age at RA onset, smoking habits, and high ACPA titers [111]. It is crucial to emphasize the significant impact of ILD on the prognosis of RA patients. Those with RA‐ILD face a mortality risk that is three times higher than RA patients without ILD. ILD plays a critical role, accounting for approximately 13% of the excess mortality observed in RA patients compared to the general population [115].

### 4.4 Risk for Developing Malignancies

A meta‐analysis of the research conducted between 1990 and 2007 has indicated that patients with RA have a slightly but significantly increased risk of developing malignancies compared to the general population [116]. This elevated risk of cancer in RA may be attributed to immunological aging associated with the condition, largely due to the presence of chronic inflammation and impaired DNA repair mechanisms [58]. Data from the National Health and Nutrition Examination Survey (NHANES) revealed that individuals with RA had a 1.63‐fold increased odds of cancer incidence [117]. Skin cancer and lymphoma are among the few specific malignancies with marginally higher incidences, contributing to this slightly increased risk of cancer. The increased risk and the type of cancer show different patterns of variability depending on the countries studied. In Japan, for example, compared with other countries, methotrexate‐associated lymphoproliferative disorders (MTX‐LPD) and lymphomas are more frequent, whereas the incidence of other types of cancer (colon, rectum, and liver) is lower [118, 119, 120].

More recent evidence suggests that RA patients treated with biologic disease‐modifying antirheumatic drugs (bDMARDs) may have a lower cancer risk compared to the general population [120]. A Taiwanese study indicated that RA patients receiving TNF inhibitors (TNFi) had a reduced risk of cancer compared to those treated with non‐bDMARDs alone [121]. However, a meta‐analysis of observational studies found no significant change in the risk of overall malignancy in patients treated with TNFi [122], and data from the UK and Spain showed unchanged cancer mortality rates among RA patients on TNFi therapy [123, 124]. Additionally, a meta‐analysis of randomized controlled trials, which included data from 63 trials and 29 423 patients, revealed no statistically significant increase in cancer risk when using biological agents compared to control groups [125]. Moreover, a real‐world multi‐database analysis examining RA patients showed no significant association between various bDMARDs or targeted synthetic (ts)DMARDs and the risk of any malignancies [126].

### 4.5 Cognitive Dysfunction and Depression

Depression is a prevalent comorbidity in RA patients and is a significant contributor to frailty. Approximately 66% of RA patients experience depression, and around 70% suffer from anxiety [127, 128, 129, 130]. A 2013 systematic review and meta‐analysis found that 16.7% of RA patients meet the diagnostic criteria for major depressive disorder [131]. Comorbid depression negatively impacts the quality of life, physical and mental health, mortality, and the severity of pain and symptoms in RA patients [132]. Depressive symptoms may also increase the likelihood of frailty by affecting behavior and activity levels, leading to increased disability and eventually frailty [133]. Sanders and colleagues demonstrated a strong correlation between depressive symptoms and pain over time, which held true regardless of age, frailty status, or follow‐up duration [134]. This relationship is sometimes referred to as the “pain‐depression dyad” [135].

Cognitive impairment is another chronic condition associated with aging and is characterized by difficulties with memory, learning, problem‐solving, concentration, or decision‐making [136]. There is a growing body of evidence linking RA to cognitive decline [137, 138]. The mechanisms underlying the association between RA and cognitive impairment are not completely understood but may involve chronic inflammation, immune system dysregulation, medication side effects, genetic predispositions, chronic pain, and mental health issues. Neuroinflammatory reactions in the brain can be caused by immune complexes, cytokines, and autoantibodies like RF. Non‐canonical signaling pathways and interactions with receptors might allow cytokines, such as TNF alpha and IL‐1, to alter neuron excitability [139, 140]. Additionally, IL‐6 has been reported to negatively impact cognition [139].

The premature immunosenescence in RA may contribute to cognitive dysfunction. Compared with healthy controls, patients with RA exhibit reduced memory performance, even after adjusting for age and depressive symptoms. Furthermore, memory abilities are inversely correlated with the expansion and activation of senescence‐associated T cells, such as CD8^+^CD28^−^ cells [141]. CD8^+^CD28^−^ T cells represent a heterogeneous population of highly differentiated lymphocytes linked to immunosenescence. Within this population, Terminally Differentiated Effector Memory T cells re‐expressing CD45RA (TEMRA) form a phenotypically defined subset, characterized by the re‐expression of CD45RA, although not all CD8^+^CD28^−^ T cells display a TEMRA phenotype. TEMRA cells show multiple features of cellular aging, including acquisition of inflammatory characteristics typical of the senescence‐associated secretory phenotype (SASP), reduced proliferative capacity, accumulation of DNA damage, and loss of telomerase activity [142].

The development of autoantibodies against brain antigens is another immune‐related factor contributing to cognitive impairment in RA. The prevalence of autoantibodies in the bloodstream increases with healthy aging and is evident early in RA disease. Consequently, immunosenescence and cognitive decline often work in tandem, potentially shortening the lifespan of elderly individuals. Moreover, cognitive impairment in LORA patients may be linked to commonly used RA medications, such as MTX and corticosteroids [143, 144, 145].

Despite the incomplete understanding of the exact mechanisms behind the link between cognitive impairment and depression in RA, it is crucial for healthcare professionals to adopt preventive measures, such as routinely assessing cognitive function in individuals diagnosed with LORA.

## 5 Challenges in the LORA Treatment

Before the widespread use of bDMARDs, RA was associated with a significantly higher mortality rate, particularly in cases of greater clinical severity [146]. Life expectancy in women and men with RA was reduced by 4 and 2 years, respectively, compared to those without RA, and these deficits persisted over the decades despite overall life expectancy improvements in RA patients aligning with the general population [147]. However, since the introduction of advanced treatments like bDMARDs, the mortality rate for RA patients has significantly decreased [148, 149].

**FIGURE 10:**
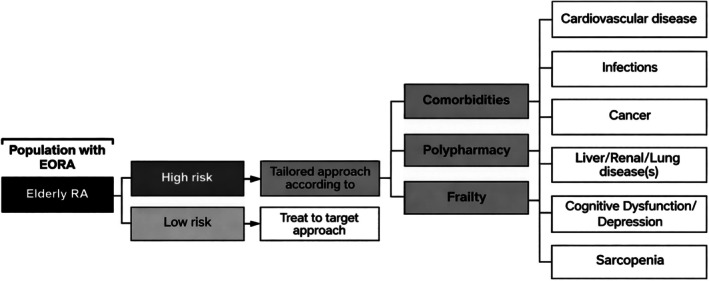
Therapeutic approach to elderly‐onset rheumatoid arthritis (LORA). Suggested customized approach based on the prevalence of multimorbidity, polypharmacy, and frailty when treating the single patient.

Managing LORA is challenging due to multimorbidity, polypharmacy, and frailty. The therapeutic approach needs to be carefully tailored, and current management methods have limitations (Figure 10). In this clinical setting, physicians should be cautious with the use of multiple medications due to the risk of drug–drug interactions, nonadherence in older adults, and potential severe consequences such as delirium, gastrointestinal bleeding, fractures, and falls [150, 151].

Biological age, which is determined by changes in biological function, plays a significant role in an individual's ability to tolerate illness, as opposed to chronological age. Patients with advanced LORA are at a higher risk of developing frailty due to a greater likelihood of experiencing functional decline, depression, cognitive impairment, falls, malnutrition, and polypharmacy [152].

Treatment of pre‐frail and frail patients with LORA should aim at returning them to a non‐frail or pre‐frail stage, respectively, to prevent progression to irreversible geriatric syndromes. Patients with LORA who are not at imminent risk of dying and who are aging well should be treated aggressively using the treat‐to‐target (T2T) approach.

In addition to pharmacological treatment, there is a need for more rheumatological rehabilitation programs for individuals with LORA. These programs should include assistive devices, physical therapy, analgesic modalities, exercise regimens, hot spring hydrotherapy, and balance exercises [153]. These comprehensive approaches can help improve the quality of life and overall outcomes for patients with LORA.

### 5.1 Treat‐to‐Target Strategies in LORA

In the T2T approach, it is generally agreed that older RA patients should be managed similarly to younger ones [154, 155, 156]. However, LORA treatment is complicated by common comorbidities and frailty, requiring tailored therapeutic intensity [157]. Treatment goals—symptom control, structural preservation, autonomy, and reduced mortality—are consistent with YORA [158, 159], though LDA may be a more realistic target than remission in LORA [160].

Despite guidelines, elderly patients often receive less intensive treatment due to concerns about drug tolerance and interactions from comorbidities [161, 162]. Age significantly affects RA drug choices [163]. US data show patients over 75 are 65%–74% less likely to receive DMARDs than younger seniors, especially if treated by non‐rheumatologists [164]. LORA patients also receive lower MTX doses, less combination therapy, and fewer biologics [165, 166, 167, 168]. Glucocorticoids are more commonly prescribed in LORA. Although MTX is used frequently, it is prescribed in lower doses due to renal impairment and comorbidities [161]. Polypharmacy increases adverse event risk, complicating decisions about aggressive treatment strategies.

Clinical evidence remains limited due to the underrepresentation of elderly patients in trials. A 3‐year study of 197 LORA patients (avg. age 74.4) using T2T found 41.6% received bDMARDs, 84.7% used MTX (avg. dose: 9.9 mg/week), with 61.1% experiencing MTX side effects; 40.2% could not receive full doses due to renal dysfunction [169].

Data from the DREAM registry and other studies suggest TNFi are slightly less effective in LORA but still significantly reduce disease activity [170, 171]. While infliximab is more common in YORA, LORA patients are more likely to receive abatacept or etanercept. Interestingly, some data suggest better disease activity improvement in LORA than in YORA with TNFi [32]. Although MTX + TNFi is effective in both groups, LORA patients have higher rates of serious adverse events [171, 172].

Genevay et al. noted that while DAS28 scores and drug discontinuation were similar, LORA patients showed less improvement in functional ability (HAQ), especially those over 75 [173]. Although older patients have higher absolute infection risks with TNFi, the relative risk is comparable to that in younger patients [174]. Glucocorticoids increase serious bacterial infection risk in a dose‐dependent way, while TNFi and MTX have similar safety profiles [175].

Evidence for biologics and targeted synthetic DMARDs (JAKi) in LORA is limited. Tocilizumab may be less effective in older patients compared to the younger, though retention and adverse event rates are similar across age groups [176]. Abatacept effectiveness has been shown to be comparable in both age groups, although in younger patients a longer drug survival time [177].

JAK inhibitors like tofacitinib and baricitinib show similar efficacy in older and younger adults [178, 179]. However, the ORAL Surveillance trial raised concerns about increased cardiovascular events and malignancies in older patients, especially smokers or those with CV risk factors [180]. Higher infection and herpes zoster rates were also noted in elderly patients on JAKi [179, 180, 181]. Lower doses of baricitinib and filgotinib are recommended for patients over 75 [182].

Although many treatments effective in YORA are also safe and beneficial in LORA, age‐related factors demand careful consideration. Individualized treatment plans are crucial, and more prospective studies are needed to guide therapy for elderly RA patients, who are often excluded from RCTs. It would also be desirable for scientific societies to pay greater attention to the treatment of LORA: to date, there is only a single consensus statement published in Japan [12].

## 6 Conclusions

LORA represents a distinct prognostic category within the spectrum of RA, characterized by high prevalence rates of multimorbidity, polypharmacy, and frailty. Compared with younger patients, those with LORA are overall at higher risk of physical decline, malignancy, and mortality [183]. Despite advancements of the last years, the understanding and management of LORA are subjects of ongoing debate. Empirical studies have highlighted that the care provided to older RA patients in clinical settings is often suboptimal. Contributing factors to this include a paucity of robust evidence, concerns regarding potential adverse effects, and the presence of comorbid conditions. The concurrent presence of RA and various other chronic illnesses complicates the management of LORA. Addressing the diverse health needs of these patients necessitates an interdisciplinary approach that is both comprehensive and judicious, balancing the risks and benefits of escalating treatment.

According to the T2T strategy, LORA patients should receive treatment akin to their younger counterparts. The T2T approach, despite a generally less favorable prognosis in patients with LORA, appears to ensure outcomes comparable to those of younger patients [184, 185]. However, the unique drug pharmacokinetics and pharmacodynamics in the elderly population necessitate vigilant monitoring of the side‐effect profile. Future research should focus on developing tailored DMARD treatments that consider multimorbidity, polypharmacy, frailty, and safety to enhance treatment efficacy and reduce reliance on glucocorticoids. Such advancements will ultimately lead to improved quality of care for patients with LORA.

## Funding

The authors have nothing to report.

## Disclosure

The authors have nothing to report.

## Ethics Statement

The authors have nothing to report.

## Consent

The authors have nothing to report.

## Data Availability

Data sharing not applicable to this article as no datasets were generated or analyzed during the current study.
